# Efficacy and safety of sodium zirconium cyclosilicate for hyperkalaemia: the randomized, placebo‐controlled HARMONIZE‐Global study

**DOI:** 10.1002/ehf2.12561

**Published:** 2020-01-15

**Authors:** Faiez Zannad, Bang‐Gee Hsu, Yoshitaka Maeda, Sug Kyun Shin, Elena M. Vishneva, Martin Rensfeldt, Stefan Eklund, June Zhao

**Affiliations:** ^1^ Université de Lorraine, Inserm, Centre d'Investigation Clinique 1433 and Centre Hospitalier Universitaire Nancy France; ^2^ Division of Nephrology Hualien Tzu Chi Hospital, Buddhist Tzu Chi Medical Foundation Hualien Taiwan; ^3^ Nephrology Division, Department of Internal Medicine JA Toride Medical Center Ibaraki Japan; ^4^ NHIS Medical Center Ilsan Hospital Goyang‐si Gyeonggi‐do South Korea; ^5^ City Clinical Hospital No. 14 Ekaterinburg Russian Federation; ^6^ Biometrics, Late‐Stage Development, Cardiovascular, Renal and Metabolism (CVRM), BioPharmaceuticals R&D AstraZeneca Gothenburg Mölndal Sweden; ^7^ AstraZeneca Gothenburg Mölndal Sweden; ^8^ AstraZeneca Gaithersburg MD USA

**Keywords:** Hyperkalaemia, Normokalaemia, Potassium, Sodium zirconium cyclosilicate

## Abstract

**Aims:**

Sodium zirconium cyclosilicate (SZC, formerly ZS‐9) is a selective K^+^ binder to treat adults with hyperkalaemia. HARMONIZE‐Global examined the efficacy and safety of SZC among outpatients with hyperkalaemia from diverse geographic and ethnic origins.

**Methods and results:**

This phase 3, randomized, double‐blind, placebo‐controlled study recruited outpatients with serum K^+^ ≥5.1 mmol/L (measured by point‐of‐care i‐STAT device) at 45 sites in Japan, Russia, South Korea, and Taiwan. Following open‐label treatment with thrice‐daily SZC 10 g during a 48 h correction phase (CP), patients achieving normokalaemia (K^+^ 3.5–5.0 mmol/L) were randomized 2:2:1 to once‐daily SZC 5 g, SZC 10 g, or placebo during a 28 day maintenance phase (MP). The primary endpoint was mean central‐laboratory K^+^ level during days 8–29 of the MP. Of 267 patients in the CP, 248 (92.9%) entered the MP. During the CP, mean central‐laboratory K^+^ was reduced by 1.28 mmol/L at 48 h vs. baseline (*P* < 0.001). During the MP (days 8–29), SZC 5 and 10 g once‐daily significantly lowered mean central‐laboratory K^+^ by 9.6% and 17.7%, respectively, vs. placebo (*P* < 0.001 for both). More patients had normokalaemia (central‐laboratory K^+^ 3.5–5.0 mmol/L at day 29) with SZC 5 (58.6%) and 10 g (77.3%) vs. placebo (24.0%), with the greatest number of normokalaemic days in the 10‐g group. The most common adverse events with SZC were mild or moderate constipation and oedema.

**Conclusions:**

Normokalaemia achieved during the CP was maintained over 28 days with SZC treatment among outpatients with hyperkalaemia.

## 1 Introduction

Hyperkalaemia is associated with an increased risk of cardiac arrhythmias and mortality.^1^, ^2^ Hyperkalaemia risk is increased with older age and among patients with chronic kidney disease (CKD), heart failure (HF), and diabetes, as well as those receiving renin–angiotensin–aldosterone system inhibitors (RAASis).^1^, ^3^ Options for outpatient management of hyperkalaemia were previously limited to non‐specific cation‐binding organic polymers, such as sodium polystyrene sulfonate and patiromer, which bind potassium (K^+^) in exchange for sodium and calcium ions, respectively.^4^ These agents have been extensively reviewed.^4^, ^5^, ^6^, ^7^, ^8^, ^9^, ^10^

Sodium zirconium cyclosilicate (SZC, formerly ZS‐9) is a selective K^+^‐binding agent approved for the treatment of hyperkalaemia in the USA and European Union.^11^, ^12^ Because of its specific binding site geometry, SZC selectively binds monovalent cations (particularly excess K^+^ and ammonium) rather than divalent cations such as calcium and magnesium.^13^ In previous clinical trials among adults with hyperkalaemia, SZC significantly reduced serum K^+^ within 1 h of administration.^14^, ^15^, ^16^ In the Kosiborod *et al*. study among 258 patients with hyperkalaemia from the USA, Australia, and South Africa, normokalaemia (K^+^ 3.5–5.0 mmol/L) was achieved by 84% of patients within 24 h and 98% within 48 h with SZC 10 g three times daily (TID) during an initial correction phase (CP).^15^ During a subsequent 29 day maintenance phase (MP), once‐daily (QD) SZC (5, 10, or 15 g) significantly reduced mean serum K^+^ compared with placebo, with a significantly greater proportion of patients maintaining normokalaemia by end of study.^15^

HARMONIZE‐Global (NCT02875834) was designed to support findings of the initial Kosiborod *et al*. trial^15^ by including a geographically and ethnically diverse population of patients with hyperkalaemia. This study aimed to determine the efficacy of QD SZC (5 or 10 g) for maintenance of normokalaemia over 28 days after normalization of K^+^ during an initial 48 h CP. Safety and tolerability of SZC were also evaluated.

## 2 Methods

This two‐phase, prospective, randomized, double‐blind, placebo‐controlled, phase 3 study was conducted across 45 investigational sites in Japan, Russia, South Korea, and Taiwan between 3 March, 2017, and 14 February, 2018 (Supporting Information, *Table* *S1*). The study was conducted in accordance with the International Conference on Harmonisation/Good Clinical Practice guidelines, and the investigation conforms with the principles outlined in the Declaration of Helsinki (Br Med J 1964; ii: 177). All patients, or their legally acceptable representatives, provided written informed consent. The study protocol and consent forms were approved by independent ethics committees for each site prior to study start.

### 2.1 Patient selection

Full inclusion and exclusion criteria are described in Supporting Information, *Table* S2. Key eligibility criteria were documented hyperkalaemia (determined by two consecutive i‐STAT K^+^ values measured 60 min apart, both ≥5.1 mmol/L), the ability to have repeat blood draws, and written informed consent. Exclusion criteria included pseudohyperkalaemia, receiving dialysis, life expectancy <3 months, pregnancy, cardiac arrhythmia requiring immediate treatment, diabetic ketoacidosis, active treatment with sodium polystyrene sulfonate or lactulose, participation in another clinical trial with an investigational product during the last 3 months prior to study entry, any condition that would affect adherence or place a patient at undue risk, and known hypersensitivity to SZC.

### 2.2 Study design

Patients received oral SZC 10 g TID for 48 h during the open‐label CP. Patients who achieved K^+^ 3.5–5.0 mmol/L, measured locally using whole blood with a point‐of‐care i‐STAT device (referred to as i‐STAT K^+^), in the morning on day 3 of the CP qualified for entry into the double‐blind 28 day dosing MP and were randomized 2:2:1 to receive SZC 5 g, SZC 10 g, or placebo QD. K^+^ was also measured by a central laboratory using serum (referred to as central‐laboratory K^+^). Patient eligibility for enrolment, randomization, and treatment decisions was based on i‐STAT K^+^ values; central‐laboratory K^+^ was used for statistical analyses. K^+^ was measured twice 60 (±10) min apart within 1 day prior to administration of the first dose; at 1, 2, and 4 h after the first dose on day 1; and prior to and 1 h after first dose on day 2 of the CP. Eligibility for randomization was based on i‐STAT K^+^ obtained on the morning of study day 3 (after 48 h of open‐label treatment).

Randomization codes were computer generated in blocks to ensure approximate balance (2:2:1) between the SZC 5 g, SZC 10 g, or placebo QD groups. Randomization was stratified by country. Patients were assigned codes by investigators via an interactive voice/web response system. The external appearance of study drug sachets was identical, but their volume differed depending upon the randomized treatment group.

After randomization, serum K^+^ was measured in the morning before the SZC dose on days 1, 2, 5, 8, 12, 15, 19, 22, 26, and in the morning on day 29 one day after the last dose of SZC on day 28 and at end of study (defined as 7 ± 1 days following last treatment dose). Day 3 of the CP was also considered day 1 of the MP. All serum samples were examined and redrawn if the i‐STAT analysis indicated any haemolysis or other artefacts.

Study treatment could be discontinued because of patient decision, adverse event (AE), severe non‐adherence to study protocol, risk to patient as determined by the investigator, pregnancy, requirement for the use of prohibited or contraindicated medications, initiation of dialysis, and change in RAASi and/or diuretic therapy or dose during the study period. Patients with severe hypokalaemia (i‐STAT K^+^ <3.0 mmol/L) at any time or hyperkalaemia (i‐STAT K^+^ >6.2 mmol/L) during the 28 day MP (confirmed by taking a second K^+^ measurement 10 ± 2 min later) discontinued study treatment and immediately received appropriate care (Supplementary Methods).

### 2.3 Study endpoints

The primary efficacy endpoint was mean central‐laboratory K^+^ level during days 8–29 of the 28 day randomized MP following treatment with SZC 5 or 10 g vs. placebo. Secondary endpoints for the CP included change from baseline in central‐laboratory K^+^, the exponential rate of change in central‐laboratory K^+^, the proportion of patients who achieved normokalaemia (defined as central‐laboratory K^+^ 3.5–5.0 mmol/L) at 24 and 48 h, and time to normalization during the 48 h period. Secondary endpoints for the MP included the proportion of patients with normokalaemia at the end of the 28 day period, the total number of days that patients were normokalaemic during the 28 day period, time to hyperkalaemia (defined as central‐laboratory K^+^ ≥5.1 mmol/L), and mean change in serum aldosterone and plasma renin. Safety was assessed by monitoring frequency of AEs and serious AEs. Oedema was evaluated by the standardized Medical Dictionary for Regulatory Activities query (SMQ) for haemodynamic oedema, effusions, and fluid overload (referred to as SMQ oedema).

### 2.4 Statistical analysis

Assuming an interpatient standard deviation of 0.50, a sample size of 255 patients (102 patients for each SZC treatment arm and 51 patients for the placebo arm) had >90% power to detect a mean difference of 0.30 mmol/L in central‐laboratory K^+^ from days 8 to 29 during the randomized MP for each SZC dose vs. placebo comparison using a two‐sided *t*‐test at a significance level of 5%. Assuming 95% of patients would have normokalaemia after open‐label treatment, ~269 patients were needed to enter the CP. All patients who entered the 48 h CP and were randomized to the 28 day MP were included in the efficacy analyses for each respective period (full analysis set). Safety analyses were conducted for patients who received ≥1 dose of study treatment in the CP or MP (safety analysis set).

If central‐laboratory K^+^ data were missing, i‐STAT K^+^ was used (adjusted to reflect the mean difference between i‐STAT and central‐laboratory K^+^ and laboratory K^+^ from available paired samples collected at the same time point).

A sequential closed testing procedure controlled the overall type I error rate at 5%. A fixed hierarchical sequence was employed (see Supporting Information, *Table* S3 for the variable test order); progression to the next test in the sequence continued until a two‐sided *P* > 0.05 was encountered (*P*‐values described as 1st, 2nd, 3rd controlled, etc.), at which point further inferential testing ceased and subsequent *P*‐values (or *P*‐values for variables not part of the closed procedure) were described as nominal. Detailed methods describing the statistical approaches are summarized in Supplementary Methods. All analyses were conducted using SAS, version 9.3.

## 3 Results

### 3.1 Study participants

**Figure 1:**
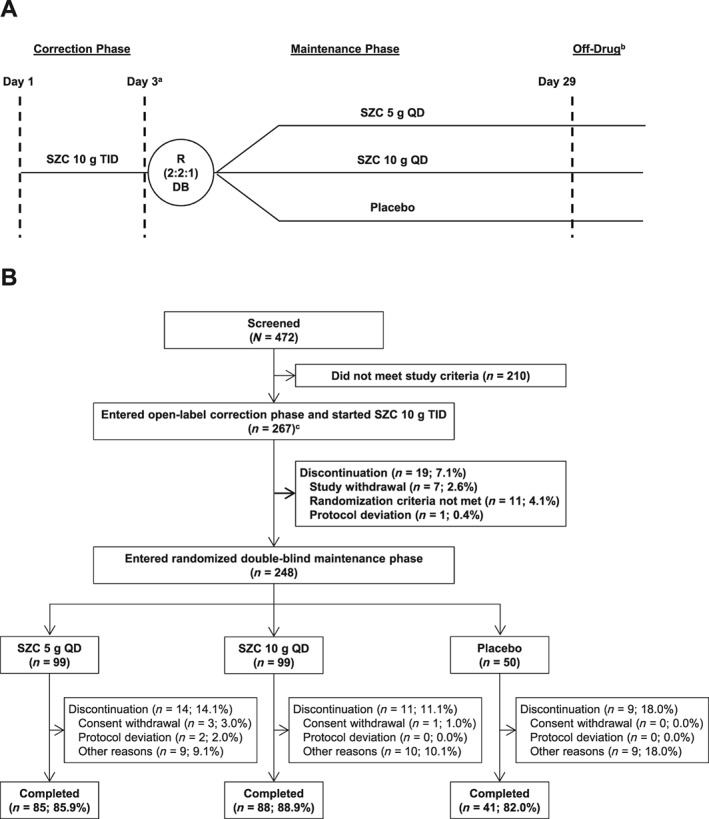
Study design (A) and patient disposition (B). ^a^Day 3 of the correction phase was also considered day 1 of the maintenance phase. ^b^Off‐drug visit occurred within 7 (±1) days after last dose administration. ^c^Five patients who did not meet the study inclusion/exclusion criteria were enrolled in the correction phase. DB, double‐blind; QD, once‐daily; R, randomization; SZC, sodium zirconium cyclosilicate; TID, thrice‐daily.

Of 472 patients screened, 262 met eligibility criteria and were enrolled in the 48 h CP (*Figure* *1*). Five additional patients were enrolled in the CP, of whom four were subsequently randomized to the MP, were later identified as having met exclusion criterion 5 (Supporting Information, *Table* S2). According to the pre‐specified intention‐to‐treat analysis, these patients were allowed to continue in the study and were included in analyses of efficacy and safety, making a total of 267 enrolled and treated patients. Of the 267 patients treated, 260 (97.4%) completed the CP. Of these 260 patients, 12 were not randomized into the MP. During the MP, 214 of 248 patients (86.3%) completed day 29 and end‐of‐study procedures.

During the CP, 11 patients had important protocol deviations prior to and during treatment, of which five used prohibited concomitant medications. During the MP, a total of 31 patients had important protocol deviations, including 13 (8, 5, and 0 from the SZC 5 g, SZC 10 g, and placebo groups, respectively) who used prohibited concomitant medications, most commonly changes in diuretics and RAASis (Supporting Information, *Table* S4). The demographics and baseline characteristics of patients randomized to receive SZC 5 g, SZC 10 g, or placebo were well balanced (*Table* 1). The study population was predominantly male (64.0%) and of Asian descent (85.0%). Most patients at CP baseline had CKD (78.3%) or diabetes (64.4%), and 18.7% had HF, with the most frequent combined co‐morbidity category being CKD and diabetes (44.2%). Many patients were receiving RAASis [204/267 (76.4%)]. Of these 204 RAASi‐treated patients, 109 (53.4%) and 69 (33.8%) were being treated at ≥50% or <50% of the maximum recommended target dose, respectively, and for 26 patients (12.7%), data on RAASi dose were missing.

**Table 1:** Patient demographics and baseline characteristics (full analysis set)

	Correction phase		Maintenance phase	
	Overall (*N* = 267)	SZC 10 g (*n* = 99)	SZC 5 g (*n* = 99)	Placebo (*n* = 50)
Age (years), mean ± SD	67.8 (10.8)	68.0 (10.2)	66.7 (11.4)	69.4 (10.3)
Age category, *n* (%)				
<55 years	31 (11.6)	12 (12.1)	13 (13.1)	4 (8.0)
55 to <65 years	69 (25.8)	19 (19.2)	30 (30.3)	14 (28.0)
≥65 years	167 (62.5)	68 (68.7)	56 (56.6)	32 (64.0)
Sex, *n* (%)				
Male	171 (64.0)	61 (61.6)	63 (63.6)	36 (72.0)
Female	96 (36.0)	28 (38.4)	36 (36.4)	14 (28.0)
Race, *n* (%)				
Asian	227 (85.0)	86 (86.9)	87 (87.9)	43 (86.0)
White	40 (15.0)	13 (13.1)	12 (12.1)	7 (14.0)
Other	0	0	0	0
Country, *n* (%)				
Japan	68 (25.5)	27 (27.3)	26 (26.3)	13 (26.0)
Korea	121 (45.3)	45 (45.5)	46 (46.5)	23 (46.0)
Russia	40 (15.0)	13 (13.1)	12 (12.1)	7 (14.0)
Taiwan	38 (14.2)	14 (14.1)	15 (15.2)	7 (14.0)
Serum K^+^ (mmol/L), mean ± SD	5.71 (0.50)	5.71 (0.50)	5.68 (0.49)	5.66 (0.45)
Serum K^+^ (mmol/L), *n* (%)				
3.5 to 5.0	14 (5.2)	4 (4.0)	7 (7.1)	3 (6.0)
5.1 to <5.5	82 (30.7)	33 (33.3)	30 (30.3)	14 (28.0)
5.5 to <6.0	96 (36.0)	32 (32.3)	36 (36.4)	21 (42.0)
≥6.0	75 (28.1)	30 (30.3)	26 (26.3)	12 (24.0)
Co‐morbidity, *n* (%)^a^				
CKD	209 (78.3)	82 (82.8)	82 (82.8)	35 (70.0)
Diabetes	172 (64.4)	66 (66.7)	67 (67.7)	29 (58.0)
Heart failure	50 (18.7)	19 (19.2)	18 (18.2)	8 (16.0)
CKD only	60 (22.5)	23 (23.2)	22 (22.2)	13 (26.0)
Diabetes only	29 (10.9)	9 (9.1)	10 (10.1)	8 (16.0)
HF only	10 (3.7)	4 (4.0)	1 (1.0)	2 (4.0)
CKD and diabetes	118 (44.2)	47 (47.5)	46 (46.5)	18 (36.0)
CKD and HF	15 (5.6)	5 (5.1)	6 (6.1)	3 (6.0)
Diabetes and HF	9 (3.4)	3 (3.0)	3 (3.0)	2 (4.0)
Diabetes, CKD, and HF	16 (6.0)	7 (7.1)	8 (8.1)	1 (2.0)
None of the above	10 (3.7)	1 (1.0)	3 (3.0)	3 (6.0)
History of hypertension, *n* (%)	225 (84.3)	85 (85.9)	83 (83.8)	41 (82.0)
RAASi use, *n* (%)	204 (76.4)	78 (78.8)	75 (75.8)	41 (82.0)
RAASi dose, *n* (%)				
≥50% of target RD	109/204 (53.4)	46/78 (59.0)	38/75 (50.7)	21/41 (51.2)
<50% of target RD	69/204 (33.8)	23/78 (29.5)	26/75 (34.7)	16/41 (39.0)
Missing dose information	26/204 (12.7)	9/78 (11.5)	11/75 (14.7)	4/41 (9.8)
Loop diuretic use, *n* (%)	75 (28.1)	29 (29.3)	31 (31.3)	12 (24.0)
Any risk factor for hyperkalaemia^b^	262 (98.1)	99 (100.0)	97 (98.0)	50 (100.0)

CKD, chronic kidney disease; HF, heart failure; RAASi, renin–angiotensin–aldosterone system inhibitor; RD, recommended dose; SD, standard deviation; SMQ, standardized Medical Dictionary for Regulatory Activities query; SZC, sodium zirconium cyclosilicate.

a Defined by SMQ narrow preferred terms, and patients counted only once per category.

b CKD, diabetes, HF, or RAASi use.

### 3.2 Efficacy

All controlled *P*‐values at each step of the sequential closed testing procedure were significant at the 0.05 level.

### 3.3 Correction phase

**Figure 2:**
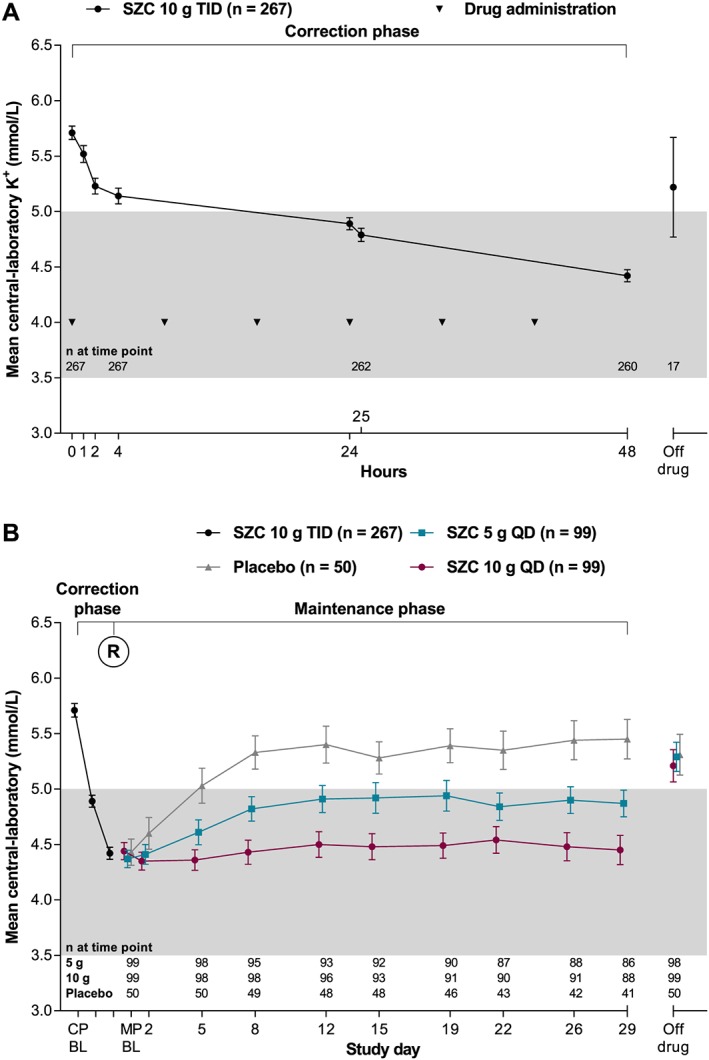
Mean central‐laboratory K^+^ over time (A) during the 48 h open‐label correction phase and (B) according to the randomized groups during the 28 day maintenance phase. Error bars are the 95% confidence interval. Grey shading indicates the normokalaemic range. BL, baseline; CP, correction phase; MP, maintenance phase; QD, once‐daily; R, randomization; SZC, sodium zirconium cyclosilicate; TID, thrice‐daily.

During the CP, when open‐label SZC 10 g TID was administered, mean central‐laboratory K^+^ was reduced relative to baseline (*Figure* *2* *A*). From a baseline mean central‐laboratory K^+^ of 5.7 mmol/L, the mean change in central‐laboratory K^+^ was −0.81 mmol/L [95% confidence interval (CI), −0.86, −0.76; nominal *P* < 0.001] at 24 h and −1.28 mmol/L (95% CI, −1.34, −1.22; 1st controlled *P* < 0.001) at 48 h. The exponential rate of change in central‐laboratory K^+^ during this period was significant (model coefficient for time −0.004; nominal *P* < 0.001). Central‐laboratory K^+^ was within the normal range in 63.3% (95% CI, 57.2%, 69.1%) of patients at 24 h and in 89.1% (95% CI, 84.8%, 92.6%) of patients at 48 h. Most patients first achieved normokalaemia 4 h after initiating SZC (Supporting Information, *Figure S1A*), and the proportion achieving normokalaemia further increased with continued dosing up to 48 h.

### 3.4 Maintenance phase

Following randomization into the 28 day MP, patients receiving SZC 5 and 10 g QD had dose‐dependently and statistically significantly lower mean central‐laboratory K^+^ during days 8–29 compared with those receiving placebo, with reductions of 9.6% and 17.7%, respectively (*Table* 2). After the initial reduction in central‐laboratory K^+^ during the CP (mean central‐laboratory K^+^: 4.4 mmol/L), patients randomized to SZC 10 g QD maintained this reduction (change from start of MP to day 29: 0.02 mmol/L), whereas among patients randomized to placebo, central‐laboratory K^+^ rose to pre‐treatment levels (change from start of MP to day 29: 1.02 mmol/L; *Figure* *2* *B*). Patients randomized to SZC 5 g QD showed partial maintenance of central‐laboratory K^+^ (change from start of MP to day 29: 0.47 mmol/L; *Figure* *2* *B*). A pre‐specified subgroup analysis was performed based on HF status at baseline. SZC maintained similar reductions vs. placebo in mean central‐laboratory K+ during days 8–29 among patients with and without HF (Supporting Information, *Table* S5). Other pre‐specified subgroup analyses by presence or absence of CKD, diabetes, or RAASi use and a post hoc subgroup analysis by age above and below the median were also consistent with the overall analysis (data not shown).

**Table 2:** Primary and secondary endpoints during the maintenance phase (full analysis set) ordered according to the sequential closed testing procedure

	SZC 10 g (*N* = 99)	SZC 5 g (*N* = 99)	Placebo (*N* = 50)
Sequential closed testing procedure endpoints			
Mean central‐laboratory K^+^ during days 8–29 (primary endpoint)^a^	*n* = 96	*n* = 95	*n* = 49
Geometric LSM (95% CI) (mmol/L)	4.38 (4.27, 4.50)	4.81 (4.69, 4.94)	5.32 (5.16, 5.49)
Geometric mean ratio (95% CI) vs. placebo	0.82 (0.80, 0.85)	0.90 (0.88, 0.93)	
2nd and 3rd controlled *P*‐values, respectively	<0.001	<0.001	
Patients remaining normokalaemic on day 29^b^	*n* = 97	*n* = 99	*n* = 50
*n* (%)	75 (77.3)	58 (58.6)	12 (24.0)
Adjusted odds ratio (95% CI) vs. placebo	18.19 (7.16, 46.21)	6.34 (2.69, 14.98)	
4th and 5th controlled *P*‐values, respectively	<0.001	<0.001	
Days normokalaemic during MP days 8–29^c^	*n* = 97	*n* = 99	*n* = 50
LSM (95% CI)	15.62 (13.37, 17.88)	10.81 (8.62, 13.00)	3.54 (0.88, 6.21)
LSM difference (95% CI) vs. placebo	12.08 (9.12, 15.04)	7.27 (4.32, 10.21)	
6th and 7th controlled *P*‐values, respectively	<0.001	<0.001	
Patients with hyperkalaemia (>5.1 mmol/L)^d^	*n* = 97	*n* = 99	*n* = 50
*n* (%) with hyperkalaemia	45 (46.4)	66 (66.7)	47 (94.0)
Adjusted hazard ratio (95% CI) vs. placebo	0.16 (0.10, 0.25)	0.44 (0.30, 0.66)	
8th and 9th controlled *P*‐values, respectively	<0.001	<0.001	
Other endpoints			
Serum aldosterone (pmol/L)^e^	*n* = 68	*n* = 76	*n* = 40
Mean change from BL at day 29 (95% CI)	−90 (−112, −68)	−103 (−151, −54)	41 (−22, 104)
Mean difference (95% CI) vs. placebo	−131 (−187, −76)	−144 (−224, −63)	
Nominal *P*‐value	<0.001	0.001	
Plasma renin, pmol/L^e^	*n* = 78	*n* = 75	*n* = 37
Mean change from BL at day 29 (95% CI)	−1.56 (−2.50, −0.62)	−0.27 (−0.51, −0.04)	−0.25 (−1.16, 0.66)
Mean difference (95% CI) vs. placebo	−1.31 (−2.80, 0.18)	−0.02 (−0.73, 0.68)	
Nominal *P*‐value	0.085	0.949	

BL, baseline; CI, confidence interval; CKD, chronic kidney disease; eGFR, estimated glomerular filtration rate; HF, heart failure; LSM, least squares mean; *N*, number in the full analysis set; *n*, number evaluable at analysis time point; RAASi, renin–angiotensin–aldosterone system inhibitor; SZC, sodium zirconium cyclosilicate.

a Back‐transformed (*e* ^value^) geometric LSM and geometric mean ratio were derived from a mixed‐effects model of log‐transformed central‐laboratory K^+^ levels. Fixed effects were treatment group, visit, treatment‐by‐visit interaction, and baseline covariates (central‐laboratory K^+^ for correction and maintenance phases, eGFR, age category, country, RAASi use, and presence of CKD, HF, and diabetes mellitus). Patient was a random effect.

b Normokalaemia was defined as central‐laboratory K^+^ 3.5–5.0 mmol/L, inclusive. Day 29 was day 29 or day of last dose of study treatment if earlier. Adjusted odds ratios were calculated as the exponential of coefficients derived from a logistic regression model with terms for treatment (not including visit and treatment‐by‐visit interaction) and baseline covariates as listed in footnote a.

c The number of normokalaemic days was calculated assuming that the time interval between assessments was normokalaemic only if both the beginning and end assessment for that time interval showed normal central‐laboratory K^+^ values. If an intermediate assessment time point was missing, the time interval was extended until the next non‐missing time point. LSM and LSM differences were derived from a linear regression model with terms for treatment (not including visit and treatment‐by‐visit interaction) and baseline covariates as listed in footnote a.

d Median time to hyperkalaemia values were derived from Kaplan–Meier analysis. Adjusted hazard ratios were derived from a Cox proportional hazards regression model with terms for treatment (not including visit and treatment‐by‐visit interaction) and baseline covariates as listed in footnote a.

e *P*‐values for mean differences were derived from a two‐sample, two‐sided *t*‐test. Tests of serum aldosterone and plasma renin were not under type I error control, and *P*‐values are thus referred to as nominal.

Because there were 13 patients who used prohibited medications during the MP, including changes in type or dose of RAASi or diuretic therapy, the inclusion of these patients, as per the pre‐specified statistical analysis plan, may have influenced the primary efficacy outcome. Therefore, we conducted an additional post hoc sensitivity analysis excluding these patients, which did not reveal differences in serum K^+^ response (Supporting Information, *Table* S6).

**Figure 3:**
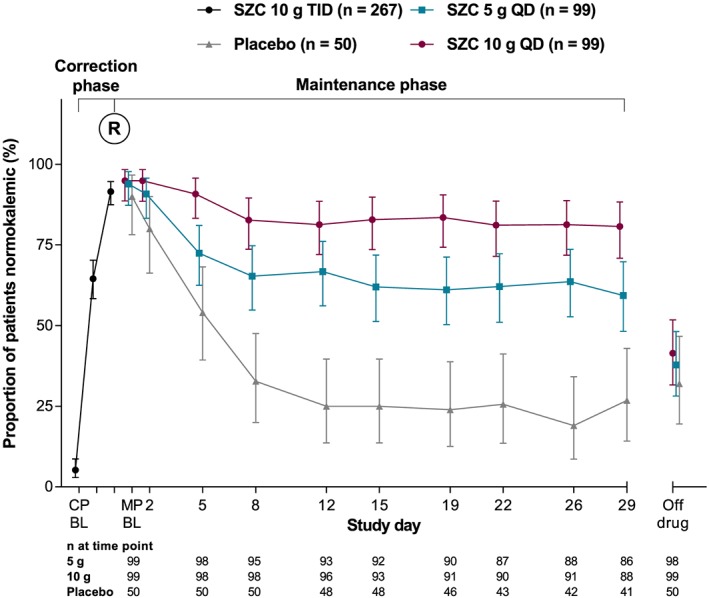
Proportion of patients with normokalaemia during the correction and maintenance phases (full analysis set). BL, baseline; CP, correction phase; MP, maintenance phase; QD, once‐daily; R, randomization; SZC, sodium zirconium cyclosilicate; TID, thrice‐daily.

The proportion of patients maintaining normokalaemia during the MP was highest among those treated with SZC 10 g QD (*Figure* *3*). For patients who received SZC 5 and 10 g QD, the odds of having normokalaemia after the last dose of study treatment were 6 and 18 times higher, respectively, than for those who received placebo, which was statistically significant (*Table* 2). Compared with placebo, SZC 5 and 10 g QD statistically significantly increased the mean number of normokalaemic days (during days 8–29 of the MP) by 7 and 12 days, respectively (*Table* 2). After randomization, there was an early, dose‐dependent separation in the proportion of patients without hyperkalaemia recurrence; more patients receiving SZC 5 and 10 g QD remained without hyperkalaemia recurrence than patients receiving placebo (Supporting Information, *Figure* S1B). Compared with placebo, the instantaneous risk of hyperkalaemia with SZC 5 and 10 g QD was significantly decreased by 56% and 84%, respectively (*Table* 2). The distributions of patients with different K^+^ values at CP baseline, CP day 3, MP day 1, and MP day 29 are shown in Supporting Information, *Figure* S2.

Mean changes in serum aldosterone showed a trend towards greater decreases from baseline for both SZC 5 and 10 g vs. placebo, with similar decreases observed in both SZC dose groups (*Table* 2). Mean changes in plasma renin showed no clear trends over time or between treatment groups (*Table* 2).

### 3.5 Treatment exposure

Treatment adherence, as measured by returned sachets, was high during both the CP (mean 99.8%; min–max 66.7–100%) and MP (mean 99.4%; min–max 75.0–145.5%). Study treatment exposure was similar across treatment groups. The SZC 5 g, SZC 10 g, and placebo groups had mean treatment exposures of 26.0, 26.4, and 26.2 days, respectively.

### 3.6 Safety

During the MP, AEs were reported by 28 (28.3%), 44 (44.4%), and 10 (20.0%) patients from the SZC 5 g, SZC 10 g, and placebo groups, respectively (*Table* 3). The frequency of serious AEs and AEs leading to discontinuation was low and was similar between treatment groups. No deaths were observed during the study.

**Table 3:** Number (%) of patients with AEs, treatment‐related AEs, serious AEs, AEs leading to discontinuation, and deaths reported during the maintenance phase (safety analysis set)

Preferred term	SZC 10 g(*n* = 99)	SZC 5 g (*n* = 99)	Placebo (*n* = 50)
AEs (occurring in ≥2 patients in any group)	44 (44.4)	28 (28.3)	10 (20.0)
Constipation	9 (9.1)	1 (1.0)	0
Diabetes mellitus	0	2 (2.0)	0
Diarrhoea	2 (2.0)	1 (1.0)	1 (2.0)
SMQ oedema^a^	15 (15.2)	5 (5.1)	0
Oedema	8 (8.1)	1 (1.0)	0
Oedema peripheral	7 (7.1)	4 (4.0)	0
Hyperkalaemia	0	3 (3.0)	2 (4.0)
Hypertension	2 (2.0)	3 (3.0)	2 (4.0)
Increased blood pressure	2 (2.0)	0	0
Upper respiratory tract infection	3 (3.0)	0	0
Ventricular extrasystoles	2 (2.0)	1 (1.0)	0
Viral upper respiratory tract infection	2 (2.0)	1 (1.0)	0
Treatment‐related AEs^b^	8 (8.1)	4 (4.0)	1 (2.0)
Bronchial obstruction	0	0	1 (2.0)
Constipation	3 (3.0)	1 (1.0)	0
Diarrhoea	1 (1.0)	0	0
Hyperkalaemia	0	1 (1.0)	0
Hypokalaemia	1 (1.0)	0	0
SMQ oedema^a^	2 (2.0)	1 (1.0)	0
Oedema	2 (2.0)	0	0
Oedema peripheral	0	1 (1.0)	0
Oedema due to renal disease	1 (1.0)	0	0
Ventricular extrasystoles	0	1 (1.0)	0
Serious AEs	3 (3.0)	4 (4.0)	1 (2.0)
Ankle fracture	0	0	1 (2.0)
Cardiac failure	1 (1.0)	0	0
Congestive cardiac failure	0	1 (1.0)	0
Cystitis	1 (1.0)	0	0
Gastritis	0	1 (1.0)	0
Hypertension	0	1 (1.0)	0
Infectious colitis	0	1 (1.0)	0
Pneumonia	0	1 (1.0)	0
Renal impairment	1 (1.0)	0	0
Upper respiratory tract infection	1 (1.0)	0	0
AEs leading to study drug discontinuation^c^	7 (7.1)	7 (7.1)	3 (6.0)
Atrial fibrillation	1 (1.0)	0	0
Bronchial obstruction	0	0	1 (2.0)
Cardiac failure	1 (1.0)	0	0
Congestive cardiac failure	0	1 (1.0)	0
SMQ oedema^a^	3 (3.0)	1 (1.0)	0
Oedema	3 (3.0)	0	0
Oedema peripheral	0	1 (1.0)	0
Oedema due to renal disease	1 (1.0)	0	0
Hyperkalaemia	0	3 (3.0)	2 (4.0)
Hypokalaemia	1 (1.0)	0	0
Pneumonia	0	1 (1.0)	0
Ventricular extrasystoles	0	1 (1.0)	0
Death	0	0	0

AE, adverse event; SMQ, standardized Medical Dictionary for Regulatory Activities query; SZC, sodium zirconium cyclosilicate.

a Preferred terms included in the oedema SMQ were ascites, fluid overload, fluid retention, generalized oedema, local swelling, oedema, oedema peripheral, pericardial effusion, pleural effusion, and pulmonary oedema.

b Based on the study investigator's causality assessment.

c Discontinuation defined as study medication stopped permanently.

Patients with multiple serious AEs or AEs leading to discontinuation of study treatment were counted once for each preferred term. AEs and serious AEs with an onset date >1 day after the last SZC dose during the correction phase, on or after the first dose during the maintenance phase, and no later than the day of last dose of study medication + 1 day were included. Preferred term defined by Medical Dictionary for Regulatory Activities (MedDRA) version 20.0.

Patients who received SZC 10 g reported a higher incidence of SMQ oedema (15.2%) and constipation (9.1%) than those who received SZC 5 g (5.1% and 1.0%, respectively) or placebo (0% for both; *Table* 3). Four oedema‐related AEs [SMQ oedema (*n* = 3) and oedema due to renal disease (*n* = 1)] were considered treatment‐related (three in the SZC 10 g group and one in the SZC 5 g group), and five oedema‐related events led to discontinuation of the study drug (four with SZC 10 g and one with SZC 5 g). All oedema‐related events were mild or moderate, and no patients were hospitalized because of HF; at the last study visit, 16 of these patients had recovered or were recovering and five had not recovered. Among patients experiencing an SMQ oedema event during the MP (*n* = 20), six (30.0% of those with oedema; 2.4% of the study population) required an increased dose or initiated a loop diuretic to treat the event, of whom four recovered and two had not recovered by the last study visit. Among patients without an SMQ oedema event during the MP (*n* = 228), 11 (4.8% of patients without oedema; 4.4% of study population) required an increased dose or initiation of a loop diuretic to treat AEs other than SMQ oedema or medical conditions. AEs of hyperkalaemia, which all led to treatment discontinuation, were reported in three patients (3.0%) who received SZC 5 g and two (4.0%) who received placebo. One AE of hypokalaemia (actual central‐laboratory K^+^ 3.1 mmol/L), which led to treatment discontinuation, occurred in the SZC 10 g group.

## 4 Discussion

The HARMONIZE‐Global study assessed the efficacy and safety of SZC for the maintenance of normokalaemia following an initial CP among outpatients with hyperkalaemia from diverse geographic and ethnic origins, 76.4% of whom were receiving RAASis. Mean central‐laboratory K^+^ levels rapidly decreased to within the normal range during the 48 h open‐label CP when all patients received SZC 10 g TID. During the randomized MP, SZC produced dose‐dependent clinically relevant and statistically significant reductions compared with placebo in mean central‐laboratory K^+^ levels during study days 8–29. During the MP, normokalaemia was maintained in greater proportions of patients for both doses of SZC vs. placebo. By the end of treatment, the number of normokalaemic days and odds of remaining in the normokalaemic range were both increased dose dependently and statistically significantly with SZC vs. placebo.

The findings of this study are consistent with those of previous clinical trials of SZC in patients with hyperkalaemia.^15^, ^16^, ^17^ The Kosiborod *et al*. study previously demonstrated significant reductions from baseline in mean serum K^+^ of −0.7 and −1.1 mmol/L with SZC 10 g TID at 24 and 48 h, respectively, that were subsequently maintained with SZC 5, 10, or 15 g QD over 28 days.^15^ Similarly, a study of 754 patients with hyperkalaemia showed significant and rapid reductions in mean central‐laboratory K^+^ levels with SZC 2.5, 5, or 10 g TID at 48 h (*P* < 0.001 vs. placebo for all three doses) that were maintained with SZC 5 or 10 g QD over 14 days.^16^

Despite the cardiovascular and renal benefits of optimal RAASi dosing, many patients receive suboptimal dosing or discontinue RAASis, often because of hyperkalaemia.^18^, ^19^ In a long‐term single‐arm open‐label study of SZC therapy over 1 year among 746 patients with hyperkalaemia, normokalaemia was maintained for up to 12 months without substantial changes in RAASi dosing.^17^ Of 483 patients using RAASis at baseline, 87% continued RAASi therapy or the dose was increased, and only 11% discontinued; and of 263 patients not using RAASis at baseline, 14% initiated RAASi therapy.^17^, ^20^ Thus, SZC may facilitate optimal RAASi dosing among patients with cardiovascular disease and/or CKD. In addition, maintenance of normokalaemia with SZC may reduce the need for dietary restrictions and improve patients' quality of life.

SZC was generally well tolerated in the current study, with low incidences of serious AEs and AEs leading to discontinuation. The most common AEs during the MP were SMQ oedema and constipation. In the current study, SMQ oedema events were reported among 15 patients (15.2%) in the SZC 10 g group and 5 (5.1%) in the SZC 5 g group during the MP. In the previous Kosiborod *et al*. study, oedema was reported more frequently with SZC 15 g than other doses or placebo; however, more patients in this dose group had HF, an estimated glomerular filtration rate <60 mL/min/1.73 m^2^, or high levels of brain natriuretic peptide at baseline.^15^ Because SZC contains sodium, whether these SMQ oedema events were related to some degree of sodium retention is unknown. However, all of these events were mild or moderate, and no patients were hospitalized for HF. In addition, these SMQ oedema events were clinically manageable, with most patients (16/20; 80%) having recovered or recovering at the last study visit either without specific treatment or by continuing, increasing, or commencing a loop diuretic with or without discontinuation of SZC.

Constipation occurred in nine patients (9.1%) in the 10 g group, one patient (1.0%) in the 5 g group, and no patients in the placebo group. These events were mild or moderate, and none led to discontinuation of study treatment. In contrast, in the previous Kosiborod *et al*. study, the incidence of constipation with SZC was numerically lower (≤2%) than that with placebo (7%).^15^ In the MP of the previous SZC dose‐ranging study (NCT01737697), constipation was reported more frequently with SZC 10 g QD vs. placebo (5% vs. 0.5%),^16^ but with a lower incidence than in the present study. It is unknown whether these between‐study differences in frequency of constipation are chance findings or relate to differences in study populations.

### 4.1 Limitations

This study has several limitations. The 29 day MP did not evaluate longer‐term efficacy and safety evaluation of SZC. Because this study was conducted in outpatient settings, patients who were hospitalized or had life‐threatening arrhythmias were excluded.

## 5 Conclusions

Normokalaemia achieved during the CP was maintained over 28 days with SZC in a geographically and ethnically diverse population of outpatients with hyperkalaemia. The most commonly reported AEs were oedema and constipation. Events of oedema recovered in the majority of patients.

## Conflict of interest

F.Z. reports consultancy, steering committee, and/or speaker fees from Amgen, AstraZeneca, Bayer, Boehringer, Boston Scientific, Cardior, CVRx, General Electric Healthcare, Janssen, Novartis, Quantum Genomics, Resmed, and Vifor‐Fresenius and also reports being founder of CardioRenal. B.G.H., Y.M., S.K.S., and E.M.V. declared no conflict of interest. M.R., S.E., and J.Z. are full‐time employees of AstraZeneca.

## Funding

This work was supported by AstraZeneca.

## Supporting information




**Table S1**. Participating study investigators and centres.
**Table S2**. Study inclusion and exclusion criteria.
**Table S3**. Sequential closed testing procedure.
**Table S4**. Important protocol deviations during the study.
**Table S5.** Subgroup analysis of mean serum K^+^ during days 8–29 by baseline heart failure status.
**Table S6**. Primary end point and sensitivity analysis excluding patients using prohibited medications during the maintenance phase (full analysis set).
**Figure S1**. (A) Time to first occurrence of normalization in central‐laboratory K+ values (Kaplan–Meier estimates; full analysis set) during the correction phase and (B) time to first recurrence of hyperkalaemia (Kaplan–Meier estimates; full analysis set) during the maintenance phase.
**Figure S2**. Distribution of central‐laboratory K^+^ during the study.
