# Supplementary material for: Efficacy and safety of sodium zirconium cyclosilicate for hyperkalaemia: the randomized, placebo‐controlled HARMONIZE‐Global study
Source: ESC Heart Fail. 2020 Jan 15;7(1):54–64. doi: 10.1002/ehf2.12561 (PMC7083449; doi:10.1002/ehf2.12561)
Supplement: Supplementary file 1 — Table S1. Participating study investigators and centres. Table S2. Study inclusion and exclusion criteria. Table S3. Sequential closed testing procedure. Table S4. Important protocol deviations during the study. Table S5. Subgroup analysis of mean serum K+ during days 8–29 by baseline heart failure status. Table S6. Primary end point and sensitivity analysis excluding patients using prohibited medications during the maintenance phase (full analysis set). Figure S1. (A) Time to first occurrence of normalization in central‐laboratory K+ values (Kaplan–Meier estimates; full analysis set) during the correction phase and (B) time to first recurrence of hyperkalaemia (Kaplan–Meier estimates; full analysis set) during the maintenance phase. Figure S2. Distribution of central‐laboratory K+ during the study. [file EHF2-7-54-s001.docx]

# Supplementary Information

**Supplementary Methods**

**Supplementary Table S1**. Participating study investigators and centres.

**Supplementary Table S2**. Study inclusion and exclusion criteria.

**Supplementary Table S3**. Sequential closed testing procedure.

**Supplementary Table S4**. Important protocol deviations during the study.

**Supplementary Table S5.** Subgroup analysis of mean serum K^+^ during days 8–29 by baseline heart failure status.

**Supplementary Table S6**. Primary end point and sensitivity analysis excluding patients using prohibited medications during the maintenance phase (full analysis set).

**Supplementary Figure S1**. (A) Time to first occurrence of normalization in central-laboratory K+ values (Kaplan–Meier estimates; full analysis set) during the correction phase and (B) time to first recurrence of hyperkalaemia (Kaplan–Meier estimates; full analysis set) during the maintenance phase.

**Supplementary Figure S2**. Distribution of central-laboratory K^+^ during the study.

## Supplementary Methods

### Criteria for treatment discontinuation and/or study withdrawal

Study treatment was immediately discontinued because of serious cardiac arrhythmias (ventricular tachycardia or ventricular fibrillation, new atrial fibrillation or atrial flutter, new paroxysmal supraventricular tachycardia [other than sinus tachycardia], new second- or third-degree atrioventricular block or significant bradycardia [defined as heart rate <40 beats/min]), acute heart failure, significant increase in the PR interval (defined as >250 ms in the absence of preexisting atrioventricular block), widening of the QRS complex (>140 ms in the absence of preexisting bundle branch block), new-onset peaked T wave, or an absolute corrected QT interval >550 ms or an increase in corrected QT interval >60 ms from baseline to >500 ms. Note that per study protocol, patients who discontinued study treatment were expected to complete the study, including the follow-up visit. Alternatively, patients who discontinued study treatment could return to the clinic 7 (± 1) days after the last study treatment administration for an end-of-study visit.

Patients were withdrawn from the study (defined as discontinuation of study treatment and assessments) at their request or because of severe nonadherence (determined by the investigator/sponsor), loss to follow-up, or death. Patients who decided to discontinue study treatment and/or assessments were asked to provide reasons for discontinuation, and the presence of adverse events, if possible, were also assessed by the investigator.

### Statistical analysis

The primary end point was analysed using a mixed-effects model with response as log-transformed central-laboratory K^+^; fixed effects as treatment group, visit (only visits on days 8–29 were included), treatment-by-visit interaction, the covariates of central-laboratory K^+^ values at correction phase and maintenance phase baselines, baseline estimated glomerular filtration rate, age category, country, baseline renin–angiotensin–aldosterone system inhibitor use, and presence of chronic kidney disease, heart failure, or diabetes; and random effect as patient. Model estimation used restricted maximum likelihood and unstructured covariance matrix. The Kenward–Roger approximation was used to estimate denominator degrees of freedom. Only central-laboratory K^+^ measurements from the days 8–29 visits were included. From the model, adjusted least squares mean central-laboratory K^+^ values averaged over days 8–29 were calculated for each treatment group and then back-transformed (exponentiated) to obtain geometric least squares mean central-laboratory K^+^ values on the original scale for each treatment group and geometric mean ratio for the differences between treatment groups.

Mean change from baseline in central-laboratory K^+^ during the correction phase was analysed using a 1-sample, 2-sided *t*-test. The exponential rate of change in central-laboratory K^+^ during the correction phase was analysed using a mixed-effects model with response as log-transformed central-laboratory K^+^. Fixed effects were similar to those for the primary end point but without the term for treatment and visit (and the interaction) and with the addition of time modelled as continuous hours; random intercept and slope (for time) were included for patient. The proportion of patients with normokalaemia at 24 and 48 hours was presented as summary statistics with 2-sided Clopper-Pearson exact confidence intervals. Time to normalization of central-laboratory K^+^ during the correction period was assessed using the Kaplan–Meier method. The odds of a patient achieving normokalaemia at day 29 (or the day of last dose of study treatment if earlier) were derived from a logistic regression model with fixed effects and baseline covariates similar to the primary end point but excluding visit and its interaction with treatment. The number of normokalaemic days was calculated using a linear regression model assuming that the time interval between assessments was normokalaemic only if both the beginning and end assessment for that time interval showed normal central-laboratory K^+^ values. If an intermediate assessment time point was missing, the time interval was extended until the next nonmissing time point (covariates were the same as those for the logistic regression analysis). Time to hyperkalaemia during the maintenance phase was assessed using Kaplan–Meier curves and adjusted hazard ratios derived from a Cox proportional hazards regression model with covariates the same as those for the logistic regression analysis. Mean differences in changes from baseline in serum aldosterone and plasma renin between treatments and at each study visit during the maintenance phase were analysed using 2-sample, 2-sided *t*-tests.

## Supplementary Tables

**Supplementary Table S1. Participating study centres and investigators**

| **Name of medical institution (department)** | **Address** | **Name of Investigator** |
| --- | --- | --- |
| Medical Corporation Tokushukai Koga General Hospital (Cardiovascular Internal Medicine) | 1555 Kounosu, 306-0041, Koga-shi, Ibaraki, Japan | Toshiyuki Takahashi |
| JA Toride Medical Center (Nephrology Medicine) | 2-1-1, Hongo, 302-0022, Toride-shi, Ibaraki, Japan | Yoshitaka Maeda |
| Hitachi, Ltd. Hitachinaka General Hospital (Internal Medicine) | 20-1 Ishikawa-cho, 312-0057, Hitachinaka-shi, Ibaraki, Japan | Akiko Onishi |
| Hanyu General Hospital (Internal Medicine) | 551 Kamiiwase, 348-8505, Hanyu-shi, Saitama, Japan | Muneo Tomizawa |
| Shizuoka Tokushukai Hospital (Internal Medicine) | 11-1 Shimogawaraminami, Suruga-ku, 421-0117, Shizuoka-shi, Shizuoka, Japan | Hiroki Yamanoue |
| Daido Clinic (Nephrology) | 8 Hakusui-cho, Minami-ku, 457-8511, Nagoya-shi, Aichi, Japan | Hideaki Shimizu |
| Toyohashi Municipal Hospital (Nephrology) | 50, Hachikennishi, Aotake–cho, 441-8570, Toyohashi-shi, Aichi, Japan | Taishi Yamakawa |
| Yao Tokushukai General Hospital (Respiratory Medicine) | 1-17 Wakakusa-cho, 581-0011, Yao-shi, Osaka, Japan | Hiromasa Harada |
| Nanbu Tokushukai Hospital (Cardiovascular Department) | 171-1 Hokama,Yaese-cho, 901-0493, Shimajiri-gun, Okinawa, Japan | Katsunori Kawamitsu |
| National Hospital Organization Chiba East Hospital (Nephrology) | 673, Nitona-cho, Chuo-ku, 260-8712, Chiba-shi, Chiba, Japan | Toshiyuki Imasawa |
| Seikeikai New Tokyo Heart Clinic (Dept of Cardiology) | 474-1, Nemoto, 271-0077, Matsudo-shi, Chiba, Japan | Yusuke Fujino |
| National Hospital Organization Kanazawa Medical Center (Renal/Collagen disease internal medicine) | 1-1, Shimoishibiki-machi, 920-8650, Kanazawa-shi. Ishikawa, Japan | Kiyoki Kitagawa |
| Ina Central Hospital (Internal Medicine) | 1313-1, Koshirokubo, 396-8555, Ina-shi, Nagano, Japan | Wataru Yumita |
| Kamiiida Daiichi General Hospital (Nephrology) | 2-70, Kamiiidakita-machi, Kita-ku, 462-0802, Nagoya-shi, Aichi, Japan | Yukari Kato |
| Seoul National University Hospital (Nephrology) | 101 Daehak-ro, Jongno-gu, 03080, Seoul, South Korea | Kwon Wook Joo |
| Sejong General Hospital (Cardiology) | 28 Hohyeon-ro, 489 beon-gil, Sosa-gu, 14754, Bucheon-si, Gyeonggi-do, South Korea | Suk Keun Hong |
| Uijeongbu St. Mary's Hospital (Nephrology) | 271 Cheonbo-ro, 11765, Uijeongbu-si, Gyeonggi-do, South Korea | Sun Ae Yoon |
| The Catholic University of Korea, Incheon St Mary’s Hospital (Nephrology) | 56 Dongsu-ro, Bupyeong-gu, 403-720, Incheon, South Korea | Seok Joon Shin |
| The Catholic University of Korea, Seoul St. Mary's Hospital (Nephrology) | 222 Banpo-daero, Seocho-gu, 06591, Seoul, South Korea | Bum Soon Choi |
| Inje University Ilsan Paik Hospital (Department of Nephrology) | 170 Juhwa-ro, Ilsanseo-gu, 10380, Goyang-si, Gyeonggi-do, South Korea | Sang Youb Han |
| Wonju Severance Christian Hospital (Nephrology) | 20 Ilsan-ro, 26426, Wonju-si, Gangwon-do, South Korea | Byoung Geun Han |
| Korea University Guro Hospital (Division of Nephrology) | 148, Gurodong-ro, Guro-gu, 08308, Seoul, South Korea | Young Joo Kwon |
| Gachon University Gil Hospital (Cardiovascular) | 21 Namdong-daero, 774beon-gil, Namdong-gu, 405-760, Incheon, South Korea | Wook-Jin Chung |
| Pusan National University Hospital (Division of Nephrology) | 179 Gudeok-ro, Seo-gu, 49241, Busan, South Korea | Sang Heon Song |
| Chonnam National University Hospital (Cardiovascular Department) | 671 Jebongro, Dong-gu, 61469, Gwangju, South Korea | Youngkeun Ahn |
| National Health Insurance Service Ilsan Hospital (Division of Nephrology) | 100, Ilsan-ro, Ilsandong-gu, 10444, Goyang-si, Gyeonggi-do, South Korea | Sug Kyun Shin |
| Hallym University Sacred Heart Hospital (Nephrology) | 22 Gwanpyeong-ro-170beon-gil, Dongan-gu, 14068, Anyang-si, Gyeonggi-do, South Korea | Sunggyun Kim |
| Hallym University Dongtan Sacred Heart Hospital (Cardiology) | 7, Keunjaebong-gil, 18450, Hwaseong-si, Gyeonggi-do, South Korea | Kyu Hyung Ryu |
| Catholic University of Korea, St Paul’s Hospital (Division of Nephrology) | 180 Wangsan-ro, Dongdaemun-gu, 02559, Seoul, South Korea | Byung Soo Kim (2017 April 10 - 2017 September 28)  Mi Jung Shin (2017 September 29 - 2018 April 27) |
| Mi Jung Shin (2017 September 29 - 2018 April 27)" |  |  |
| The Catholic University of Korea, St. Vincent’s Hospital (Nephrology) | 93 Jungbu-daero, Paldal-gu, Suwon-si, Gyeonggi-do, South Korea | Hyung Wook Kim |
| SMG - SNU Boramae Medical Center (Division of Nephrology) | 20, Boramae-ro, 5-gil, Dongjak-gu, 07061, Seoul, South Korea | Chun Soo Lim |
| Chungbuk National University Hospital (Department of Cardiology) | 776 1sunhwan-ro, Seowon-gu, 28644, Cheongju-si, Chungcheongbuk-do, South Korea | Myeong-Chan Cho |
| The Catholic University, Bucheon St Mary's Hospital (Department of Nephrology) | 327, Sosa-ro, Wonmi-gu, 14647, Bucheon-si, Gyeonggi-do, South Korea | Ho Cheol Song |
| Bessalar Clinic | Karamyshevskaya nab. 44, 123423, Moscow, Russian Federation | Svetlana Erofeeva |
| St. Petersburg City Multiservice Hospital #2 | 5 Uchebniy per. 194354, Saint-Petersburg, Russian Federation | Yury Didenko |
| City Hospital #38 n.a. N.A.Semashko | 7/2, Gospitalnaya str., 196601, Saint Petersburg, Russian Federation | Rostislav Nilk |
| City Clinical Hospital 14 | 15A, 22nd Partsiezda St., 620039, Ekaterinburg, Russian Federation | Elena Vishneva |
| Karpovich City Clinical Hospital | 17 Kurchatova ul., 660062, Krasnoyarsk, Russian Federation | Maria Rossovskaya |
| Yaroslavl Regional Clinical Hospital | 7, Yakovlevskaya str., 150062, Yaroslavl, Russian Federation | Tatyana Abissova |
| National Taiwan University Hospital | No 7 Chung Shan South Road, ROC 100, Taipei, Taiwan | Chih-Kang Chiang |
| Chung Shan Medical University Hospital | No.110, Sec. 1, Jianguo N. Rd., South Dist, Taichung, Taiwan | Horng-Rong Chang |
| Taichung Veterans General Hospital | 1650 Taiwan, Boulevard Sect. 4, 40705, Taichung, Taiwan | Ming-Ju Wu |
| Tzu Chi General Hospital | No. 707 Section 3 Chung-Yang Road, 970, Hualien City, Taiwan | Bang-Gee Hsu |
| Veterans General Hospital -Taipei | No. 201, Sec. 2, Shih-Pai Rd, Taipei, Taiwan | Wei-Cheng Tseng |

**Supplementary Table S2. Study inclusion and exclusion criteria**

| **Inclusion Criteria** | **Exclusion Criteria** |
| --- | --- |
| 1. Provide informed consent 2. Adult outpatients (aged ≥18 and ≤90 years) 3. Two consecutive i-STAT K^+^ values ≥5.1 mmol/L, when measured 60 (± 10) minutes apart and within 1 day of receiving the first sodium zirconium cyclosilicate (SZC) dose 4. Ability to have repeated blood draws or effective venous catheterization 5. Female patients must either be postmenopausal for 1 year, surgically sterile, or using an acceptable method of contraception (defined as a barrier method in conjunction with a spermicide) during the study and for 3 months after the last dose of study treatment to prevent pregnancy | 1. Involvement in the planning and/or conduct of the study 2. Participation in another clinical study with an investigational product during the last 3 months 3. Pseudohyperkalaemia characterized by haemolysed blood specimen due to excessive fist clenching to make veins prominent, difficult or traumatic venipuncture, or history of severe leucocytosis or thrombocytosis 4. Use of lactulose, rifaximin, or other nonabsorbed antibiotics for hyperammonaemia 5. Use of resins (such as sevelamer acetate or sodium polystyrene sulfonate), calcium acetate, calcium carbonate, or lanthanum carbonate within 7 days prior to the first dose of study drug 6. Life expectancy of <3 months 7. Severely physically or mentally incapacitated and who, in the opinion of investigator, are unable to perform the tasks associated with the protocol 8. Women who are pregnant, lactating, or planning to become pregnant 9. Diabetic ketoacidosis 10. Presence of any condition which, in the opinion of the investigator, places the patient at undue risk or potentially jeopardizes data quality 11. Known hypersensitivity or previous anaphylaxis to SZC or its components 12. Cardiac arrhythmias requiring immediate treatment 13. Receiving dialysis 14. Plan to donate blood during the study and 3 months following the last SZC dose |

**Supplementary Table S3. Sequential closed testing procedure**

| **Sequence** | **Study Phase** | **Efficacy Variable** | **Comparison** |
| --- | --- | --- | --- |
| 1 | Correction^a^ | 48-hour open-label phase mean change from baseline of central-laboratory K^+^ 48 hours after first dose of SZC 10 g | 48 hours vs baseline |
| 2 | Maintenance^b^ | 28-day randomized treatment study phase days 8–29 mean central-laboratory K^+^ (primary end point) | SZC 10 g QD vs placebo |
| 3 | Maintenance | 28-day randomized treatment study phase days 8–29 mean central-laboratory K^+^ (primary end point) | SZC 5 g QD vs placebo |
| 4 | Maintenance | Proportion of patients who remain having normokalaemia (as defined by central-laboratory K^+^ between 3.5 and 5.0 mmol/L, inclusive) during the 28-day randomized treatment study phase at study day 29/exit^c^ | SZC 10 g QD vs placebo |
| 5 | Maintenance | Proportion of patients who remain having normokalaemia (as defined by central-laboratory K^+^ between 3.5 and 5.0 mmol/L, inclusive) during the 28-day randomized treatment study phase at study day 29/exit^c^ | SZC 5 g QD vs placebo |
| 6 | Maintenance | Number of days patients remain having normokalaemia during the 28-day randomized treatment study phase days 8 to 29, inclusive | SZC 10 g QD vs placebo |
| 7 | Maintenance | Number of days patients remain having normokalaemia during the 28-day randomized treatment study phase days 8 to 29, inclusive | SZC 5 g QD vs placebo |
| 8 | Maintenance | Time to hyperkalaemia (defined as central-laboratory K^+^ ≥5.1 mmol/L during the 28-day randomized treatment study phase) | SZC 10 g QD vs placebo |
| 9 | Maintenance | Time to hyperkalaemia (defined as central-laboratory K^+^ ≥5.1 mmol/L during the 28-day randomized treatment study phase) | SZC 5 g QD vs placebo |

QD, once daily; SZC, sodium zirconium cyclosilicate.

^a^ Correction phase refers to the 48-hour open-label initial phase. ^b^ Maintenance phase refers to the 28-day randomized treatment phase. ^c^ Study day 29/exit refers to day of last dose of study treatment.

**Supplementary Table S4. Important protocol deviations during the study**

|  | Correction Phase | Maintenance Phase | | |
| --- | --- | --- | --- | --- |
|  | **Overall**  **(N = 267)** | **SZC 5 g**  **(n = 99)** | **SZC 10 g**  **(n = 99)** | **Placebo**  **(n = 50)** |
| ≥1 important protocol deviation, n (%)^a^ | 11 (4.1) | 14 (14.1) | 14 (14.1) | 3 (6.0) |
| Did not fulfil eligibility criteria | 5 (1.9) | 2 (2.0) | 1 (1.0) | 1 (2.0) |
| Protocol-required procedure not adhered to | 3 (1.1) | 1 (1.0) | 2 (2.0) | 2 (4.0) |
| Study drug dosing error^b^ | 0 | 3 (3.0) | 6 (6.1) | 0 |
| Received prohibited concomitant medication | 5 (1.9) | 8 (8.1) | 5 (5.1) | 0 |
| Acetazolamide | 0 | 1 (1.0) | 0 | 0 |
| Aliskiren fumarate | 0 | 0 | 1 (1.0) | 0 |
| Amlodipine and valsartan | 0 | 1 (1.0) | 1 (1.0) | 0 |
| Bixalomer | 1 (0.4) | 0 | 1 (1.0) | 0 |
| Calcium acetate | 1 (0.4) | 0 | 0 | 0 |
| Calcium polystyrene sulfonate | 1 (0.4) | 1 (1.0) | 0 | 0 |
| Ferric citrate | 0 | 0 | 1 (1.0) | 0 |
| Furosemide | 0 | 3 (3.0) | 0 | 0 |
| Hydrochlorothiazide | 0 | 0 | 1 (1.0) | 0 |
| Irbesartan | 0 | 1 (1.0) | 0 | 0 |
| Olmesartan medoxomil | 1 (0.4) | 1 (1.0) | 0 | 0 |
| Spironolactone | 0 | 1 (1.0) | 1 (1.0) | 0 |
| Valsartan | 1 (0.4) | 0 | 1 (1.0) | 0 |
| Study drug adherence <80% or >120% | 1 (0.4) | 1 (1.0) | 1 (1.0) | 0 |

SZC, sodium zirconium cyclosilicate.

^a^ Occurred before the start of and/or during treatment. ^b^ Patients who had study drug dosing errors were patients whose dosing frequency was changed from once daily (QD) to once every other day (QoD) because of i-STAT K^+^ between 3.0 and 3.4 mmol/L but who were then switched back to QD dosing when, according to the protocol, these patients should have continued on QoD dosing for the remainder of the study.

Patients may have >1 important protocol deviation.

**Supplementary Table S5. Subgroup analysis of mean serum K^+^ during days 8–29 by baseline heart failure status. (full analysis set).**

|  | **SZC 10 g (N = 99)** | **SZC 5 g (N = 99)** | **Placebo (N = 50)** |
| --- | --- | --- | --- |
| **Patients with heart failure at baseline** | | | |
| n | 19 | 16 | 8 |
| Geometric LSM (95% CI), mmol/L^a^ | 4.38 (4.17, 4.60) | 4.76 (4.53, 5.00) | 5.25 (4.91, 5.61) |
| **Patients without heart failure at baseline** | | | |
| n | n = 77 | n = 79 | n = 41 |
| Geometric LSM (95% CI), mmol/L^a^ | 4.41 (4.29, 4.53) | 4.85 (4.73, 4.98) | 5.37 (5.20, 5.55) |
| ***P*-value for interaction effect between treatment and heart failure status** | 0.700 | 0.949 |  |

CI, confidence interval; CKD, chronic kidney disease; eGFR, estimated glomerular filtration rate; HF, heart failure; LSM, least squares mean; N, number in the full analysis set; n, number evaluable at analysis time point; RAASi, renin–angiotensin–aldosterone system inhibitor; SZC, sodium zirconium cyclosilicate.

^a^ Back-transformed (e^value^) geometric LSM and geometric mean ratio were derived from a mixed-effects model of log-transformed central-laboratory K+ levels. Fixed effects were treatment group, visit, treatment-by-visit interaction, baseline covariates (central-laboratory K+ for correction and maintenance phases, eGFR, age category, country, RAASi use, and presence of CKD, HF, and diabetes mellitus), and treatment by HF status interaction. Patient was a random effect.

**Supplementary Table S6. Primary end point and sensitivity analysis excluding patients using prohibited medications during the maintenance phase (full analysis set).**

|  | **SZC 10 g (N = 99)** | **SZC 5 g (N = 99)** | **Placebo (N = 50)** |
| --- | --- | --- | --- |
| **Primary endpoint** | | | |
| Mean central-laboratory K^+^ during days 8–29 (primary end point)^a^ | n = 96 | n = 95 | n = 49 |
| Geometric LSM (95% CI), mmol/L | 4.38 (4.27, 4.50) | 4.81 (4.69, 4.94) | 5.32 (5.16, 5.49) |
| Geometric mean ratio (95% CI) vs placebo | 0.82 (0.80, 0.85) | 0.90 (0.88, 0.93) |  |
| *P* value | <0.001 | <0.001 |  |
| **Excluding patients using prohibited medications** | | | |
| Mean central-laboratory K^+^ during days 8–29 (sensitivity analysis)^a^ | n = 91 | n = 89 | n = 49 |
| Geometric LSM (95% CI), mmol/L | 4.39 (4.27, 4.52) | 4.82 (4.68, 4.95) | 5.33 (5.16, 5.51) |
| Geometric mean ratio (95% CI) vs placebo | 0.82 (0.80, 0.85) | 0.90 (0.87, 0.93) |  |
| *P* value | <0.001 | <0.001 |  |

CI, confidence interval; CKD, chronic kidney disease; eGFR, estimated glomerular filtration rate; HF, heart failure; LSM, least squares mean; N, number in the full analysis set; n, number evaluable at analysis time point; RAASi, renin–angiotensin–aldosterone system inhibitor; SZC, sodium zirconium cyclosilicate.

^a^ Back-transformed (e^value^) geometric LSM and geometric mean ratio were derived from a mixed-effects model of log-transformed central-laboratory K+ levels. Fixed effects were treatment group, visit, treatment-by-visit interaction, and baseline covariates (central-laboratory K+ for correction and maintenance phases, eGFR, age category, country, RAASi use, and presence of CKD, HF, and diabetes mellitus). Patient was a random effect.

## Supplementary Figures

**Supplementary Figure S1.** (A) Time to first occurrence of normalization in central-laboratory K^+^ values (Kaplan–Meier estimates; full analysis set) during the correction phase and (B) time to first recurrence of hyperkalaemia (Kaplan–Meier estimates; full analysis set) during the maintenance phase. Grey bands are the 95% confidence interval. Normalization was defined as central-laboratory K^+^ levels between 3.5 and 5.0 mmol/L, inclusive. Hyperkalaemia was defined as central-laboratory K^+^ level ≥5.1 mmol/L.

**Supplementary Figure S2.** Distribution of central-laboratory K^+^ during the study. Grey shading indicates the normokalaemic range.
